# Free CA125 promotes ovarian cancer cell migration and tumor metastasis by binding Mesothelin to reduce DKK1 expression and activate the SGK3/FOXO3 pathway

**DOI:** 10.7150/ijbs.52097

**Published:** 2021-01-14

**Authors:** Qianyu Huo, Chen Xu, Yanhong Shao, Qin Yu, Lunhui Huang, Yunde Liu, Huijing Bao

**Affiliations:** 1 School of Medical Technology, Tianjin Medical University, Tianjin 300203, China.; 2 Laboratory Science Department, Tianjin 4th Central Hospital, Tianjin, 300100, China.; 3 Integrative Medical Diagnosis Laboratory, Tianjin Nankai Hospital, Tianjin, 300100, China; School of Medical Technology, Tianjin Medical University, Tianjin 300203, China.

**Keywords:** CA125, DKK1, MSLN, therapy, ovarian cancer

## Abstract

**Objective:** CA125/MUC16 is an O-glycosylated protein that is expressed on the surfaces of ovarian epithelial cells. This molecule is a widely used tumor-associated marker for diagnosis of ovarian cancer. Recently, CA125 was shown to be involved in ovarian cancer metastasis. The purpose of this study was to investigate the mechanism of CA125 during ovarian cancer metastasis.

**Methods:** We analyzed the Oncomine and CSIOVDB databases to determine the expression levels of DKK1 in ovarian cancer. DKK1 expression levels were upregulated or downregulated and applied with CA125 to Transwell and Western blot assays to ascertain the underlying mechanism by which CA125 stimulates cell migration via the SGK3/FOXO3 pathway. Anti-mesothelin antibodies (anti-MSLN) were used to block CA125 stimulation. Then the expression levels of DKK1were tested by enzyme-linked immunosorbent assay (ELISA) to eliminate the blocking effect of anti-MSLN to CA125 stimulation. Xenograft mouse models were used to detect the effects of CA125 and anti-MSLN on ovarian cancer metastasis *in vivo*.

**Results:** DKK1 levels were downregulated in ovarian tumor tissues according to the analyses of two databases and significantly correlated with FIGO stage, grade and disease-free survival in ovarian cancer patients. DKK1 levels were downregulated by CA125 stimulation *in vitro*. Overexpression of DKK1 reversed the ability of exogenous CA125 to mediate cell migration by activating the SGK3/FOXO3 signaling pathway. Anti-MSLN abrogated the DKK1 reduction and increased the apoptosis of ovarian cancer cells. The use of anti-MSLN in xenograft mouse models significantly reduced tumor growth and metastasis accelerated by CA125.

**Conclusions:** These experiments revealed that the SGK3/FOXO3 pathway was activated, wherein decreased expression of DKK1 was caused by CA125, which fuels ovarian cancer cell migration. Mesothelin is a potential therapeutic target for the treatment of ovarian cancer metastasis.

## Introduction

Ovarian cancer (OC) is a highly aggressive tumor and the most fatal of all common gynecological malignancies in women worldwide 1. One of the main reasons for the high mortality is that patients cannot be diagnosed before an advanced stage. The 5-year survival among women diagnosed with OC is 46.2%. Over 70% of OC is not diagnosed before progressing to stage III or IV 2. In epithelial ovarian cancer, late-stage disease has a 5-year relative survival rate of 29%, in contrast to 92% for early-stage disease 3. Tumors likely invade adjacent organs or metastasize to the peritoneal cavity at an early stage in progression 4. According to the staging classification of ovarian cancer, extension or metastasis to extraovarian/extratubal pelvic organs is defined as stage II, metastasis to the retroperitoneal lymph nodes is defined as stage III, and distant metastasis is defined as stage IV 5. Thus, in ovarian cancer, tumor invasion and metastasis play a decisive role in the progression of disease.

An abnormal level of CA125 was observed in 99% of serous carcinoma cases rated I to IV in FIGO staging 6. CA125 (MUC16) is currently a well-established serum tumor marker for ovarian epithelial cancer and may diagnose cancer at an early stage 7,8. Only 1 percent of healthy persons and 6 percent of patients with nonmalignant disease had serum CA125 levels above 35 U per milliliter. However, 82 percent of surgically demonstrated ovarian cancer patients had elevated levels of CA125, and among those, levels of CA125 correlated with progression or regression of disease in 93 percent of the cases 9. In postoperative patients who underwent cytoreductive or radical surgery and chemotherapy, serum CA125 values could indicate a relative risk of recurrence 10,11. The glycoprotein carrying the CA125 antigen is encoded by the mucin 16 gene in humans 12. Mucins (MUCs) are large and heavily glycosylated proteins with a high carbohydrate content and are found at the cell surface of many epitheliums 13,14. MUC16 is a transmembrane type of mucin that is expressed on the cell surface and provides both barrier and signaling functions 15. Soluble proteolytic fragments named CA125 are released into the extracellular space and detected in the serum. CA125 has a relatively high concentration throughout the process of OC 16. Recently, an increasing number of studies have described the biological role of MUC16 in the progression and metastasis of ovarian tumors 17. The expression of MUC16 increases ovarian cancer cell motility, invasiveness, and metastatic properties and enhances tumor growth and metastases of ovarian cancer in SCID mice 18.

In our previous study, we examined whether CA125 promotes ovarian cancer cell migration and whether DKK1 reverses the ability of CA125 to induced migration. We also speculated that the serum CA125 concentration could serve as an effective index of metastasis in ovarian cancer 19. The present study was designed to determine the possible mechanism of CA125 in the metastasis of ovarian cancer. CA125 enhanced cell migration by reducing DKK1 expression and activating the SGK3/FOXO3 signaling pathway. Furthermore, by blocking the binding of free CA125 and mesothelin (MSLN) on the cell membrane, the decrease in DKK1 can be reversed. Applying a mouse monoclonal antibody targeting MSLN in mice bearing ovarian cancer cell xenografts significantly suppressed tumor growth and tumor lymphatic drainage. Our results demonstrated that CA125 via the MSLN/DKK1/SGK3/FOXO3 pathway accelerates cell migration, and targeting mesothelin may potentially be utilized in ovarian cancer therapy.

## Materials and methods

### Gene expression and survival analysis in databases

CSIOVDB (http://csiovdb.mc.ntu.edu.tw/CSIOVDB.html) is a database established for the study of ovarian cancers, including transcriptomic microarray data from 3,431 human ovarian cancer tissues with clinicopathological parameters 20. Here, the database was used to investigate the expression of DKK1 in ovarian cancer tissues and its correlation with pathological grade and clinical outcome.

DKK1 mRNA expression in ovarian cancer was investigated in the Oncomine Cancer Profiling Database (https://www.oncomine.org/) (Compendia Bioscience, Ann Arbor, MI). The filter was set as follows: gene as DKK1; analysis type as ovarian cancer vs. normal; data type as mRNA. The results are presented as box plots.

### Cell lines, antibodies and other reagents

A2780, CaOV-3, OVCAR3, and skov-3 cell lines were purchased from the American Type Culture Collection (ATCC). A2780, CaOV-3, and skov-3 cell lines were maintained in RPMI 1640 medium (Gibco, Invitrogen, CA, USA) supplemented with 10% fetal bovine serum (FBS) (Gibco, Invitrogen, CA, USA). The OVCAR3 cell line was maintained in RPMI 1640 medium supplemented with 20% FBS and 1% bovine insulin (Sigma-Aldrich, St. Louis, MO, USA). Cells were incubated at 37°C in a humidified incubator containing 5% CO_2_ in air. Rabbit anti-Akt (1:1000) (#92725), rabbit anti-p-Akt (1:1000) (#4060), and rabbit anti-PI3K (1:500) (#4292) antibodies were all purchased from Cell Signaling Technology (Danvers, MA, USA). Rabbit anti-SGK3 (1:1000) (bs-6475R) antibody was purchased from Bioss (Beijing, China). Rabbit anti-FOXO3A (1:2000) (ab12162) and Rabbit anti-LYVE-1 (1:300) (ab 14917) was purchased from Abcam (Cambridge, UK). Anti-GAPDH (1:2000) (AF7021) and anti-Tubulin-β (1:2000) (T0023) antibodies were purchased from Affinity Biosciences (Jiangsu, China). Mouse-anti-rabbit immunoglobulin G (sc-2357) (1:1000), mouse IgGκ binding protein-HRP (1:1000) (sc-516102) and mouse anti-mesothelin (1:50) (sc-365324) antibodies were purchased from Santa Cruz Biotechnology, Inc (Texas, USA). ECL Western Blotting Detection Reagents were obtained from Pierce (Texas, USA). Lipo 2000 transfection reagent was purchased from Thermo Fisher Scientific (CA, USA). Recombinant human CA125/MUC16 was purchased from R&D Systems (Minneapolis, MN).

### Treatment with CA125 and anti-MSLN antibody

Ovarian cancer cells were incubated with 0, 0.2, and 0.4 μg/mL recombinant human CA125 for 48 h as previously described 19. For further blocking assays, cells were treated with anti-MSLN monoclonal antibody at a dilution ratio of 1:50 (4 μg/mL). Two days later, the cells treated with the corresponding treatments were washed with phosphate buffered saline (PBS) or harvested for assays accordingly.

### RNA extraction, reverse transcription and quantitative PCR

RNA was isolated from OC cell lines using TRIzol reagent (Ambion, USA) according to the manufacturer's instructions. Reverse transcription was performed using a FastQuant RT Kit (Tiangen Biotech, China). Quantitative PCR reactions were conducted with SYBR Green PCR Master Mix Reagents (Tiangen Biotech, China) on the Stratagene MX3005P system. The fold change (2^-△△Ct^) in expression was calculated for the gene of interest relative to the internal control gene (GAPDH). The primer sequences used in PCRs were as follows: GAPDH: sense: 5'-CCT CCA AGG AGT AAG ACC CC-3', antisense: 5'-AGG GGT CTA CAT GGC AAC TG-3'. The primer sequences for DKK1 (Hs-QRP-40337) were purchased from GeneCopoeia, China.

### Immunoblot analysis

Cells were washed and lysed in loading buffer (KeyGEN BioTECH, China). Proteins were separated by ultrasonic and then resolved by SDS-PAGE, transferred to polyvinylidene fluoride (PVDF, 0.45 μm, Millipore, Germany) membranes and detected using specific primary antibodies, appropriate HRP-conjugated secondary antibodies and an ECL detection system. The nuclear/cytoplasmic extracts were prepared using the nuclear and cytoplasmic protein extraction kit (P0028, Beyotime, China) as per the manufacturer's instructions. Next, ImageJ software was used for densitometric analysis of the bands.

### Transient transfection

The DKK1 DNA fragments were cloned into the pEGFP-N1 plasmid (Tongyong, China). The plasmids were extracted from Escherichia coli DH5α transformed cultures using Endotoxin free Plasmid Extraction Kit (DP117, Tiangen Biotech, China) as per the manufacturer's instructions. The pEGFP-N1and pEGFP-N1-DKK1 plasmids (2.5 μg/mL) were transfected into logarithmic growth phase ovarian cancer cells using Lipofectamine 2000 (Invitrogen, CA, USA).

For knockdown of DKK1 expression, siRNA (GenePharma, China) was used in the ovarian cancer cell line. Cells were seeded into the plate, cultured for 24 h and transfected with 75 pM negative control (NC) RNA or siRNA using Lipofectamine 2000 according to the manufacturer's instructions. The siRNA sequences were sense: 5'- GCCGGAUACAGAAAGAUCATT-3' and antisense: 5'- UGAUCUUUCUGUAUCCGGCTT -3'.

After 24 h transfection, Western blots, qPCR, ELISAs and further experiments were conducted.

### ELISA

Cell culture medium was pipetted into tube and centrifugated for achieving supernatant. Determination of DKK1 in culture medium was performed using a commercial ELISA kit (EK0867) (Boster, China) according to the manufacturer's instructions. The measured intensity of the developing color was proportional to the concentration of DKK1. The O.D. absorbance was read with a microplate reader (Synergy2, USA) at 450 nm. The results are presented as O.D. levels.

### Transwell assays

The cells that were transiently transfected were harvested and seeded in transwell inserts (polycarbonate filter with 8 μm pores, Costar, USA). After overnight incubation, medium containing different concentrations of CA125 was added to observe the changes in cell migration. After 48 h of culture at 37°C, cells that remained inside the upper chamber were removed softly with a cotton swap. Cells that migrated to the lower surface of the filter membrane were fixed with anhydrous methanol and stained with 0.1% crystal violet. Filters were air dried, and photos were taken with an inverted phase contrast microscope (Nikon Eclipse Ti-U, Japan). The invading cells in 4 fields were counted, and statistical analysis was performed.

### Transcriptome sequencing analyses of CA125-treated OC cells

A total of 5×10^5^ A2780 cells were seeded per well in 6-well plates. After overnight incubation, the culture medium was changed to medium with or without CA125 (0 μg/mL or 0.2 μg/mL). The cells were incubated for 48 h, harvested in TRI-Zol and reverse transcribed. cDNA libraries were transcriptome sequenced on an Illumina HiSeq platform with paired-end 150 bp read length. Screening of differentially expressed genes was based on Poisson distribution methods. Differentially expressed genes were used for subsequent pathway analysis.

### Apoptosis detection and flow cytometry

The percent of cells that underwent apoptosis was detected by using the FITC Annexin-V and PI detection kit (KGA107, KeyGEN BioTECH, China). The procedure was performed according to the manufacturer's instructions. Briefly, the cells (approximately 1×10^5^ cells per tube) were harvested and washed and suspended in binding buffer provided by the manufacturer containing 5 μL of FITC Annexin-V and 5 μL of PI for 5 to 15 min at room temperature in the dark. The cells were then vortexed adequately and analyzed by FACS Verse (BD Biosciences, Franklin Lakes, NJ, USA) within 1 h. The data were analyzed by FlowJo (De Novo Software, Glendale, CA, USA). Ten thousand events were analyzed in each run.

### *In vivo* xenograft models and histological analysis

All animal experimental procedures were approved by the Animal Care and Welfare Committee of Tianjin Medical University. A2780 or OVCAR3 cell pellets were suspended in PBS and injected subcutaneously on right back into NOD-SCID-IL2rg mice (6-8 weeks old, female) with 5×10^6^ cells per mouse. In the experiment to verify the effect of CA125 on metastasis, the mice were injected with PBS, CA125 (0.2 μg per mouse) via the tail vein after the tumors were palpable. The mice were sacrificed after three injections when the tumors were approaching the scope of ethical approval. In the treatment experiment, the mice were injected with PBS, CA125 (0.2 μg per mouse) three times follow the cell seeded. Once the tumors were palpable, anti-mesothelin antibody (2 mg per mouse) were injected four times via the tail vein in the mice which injected CA125 before.

Tumors were measured with calipers every two days in two directions, and the tumor volume was calculated using the formula (width^2^ × length)/2. Evan's blue was injected around the tumor 2 h before the mice were sacrificed. After the animals were sacrificed, the tissues were separated. The tumors, axillary lymphatic tissues, and inguinal lymphatic tissues were fixed in 4% paraformaldehyde, embedded in paraffin and sectioned for hematoxylin and eosin (H&E) staining and immunohistochemistry (IHC) analysis.

### Statistics

Statistical analyses were performed using GraphPad Prism 6.0 Software. Student's t-test was used to compare the differences between two groups. One-way ANOVA was used to compare the differences among more than two groups. The results are presented as the mean±SD. A p value of less than 0.05 was considered statistically significant.

## Results

### DKK1 is downregulated in ovarian cancer tissue

**Figure 1:**
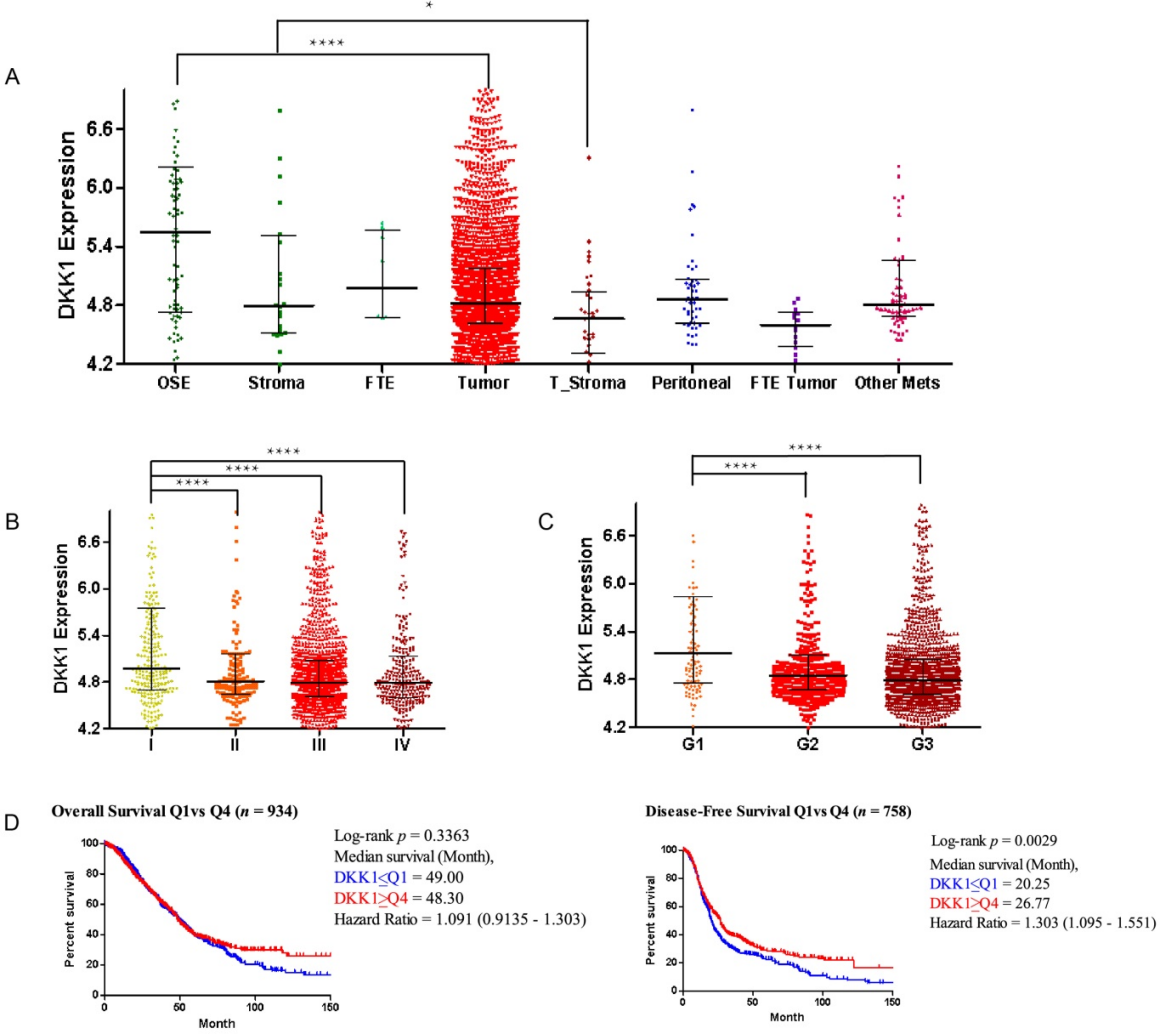
** DKK1 expression levels in the CSIOVDB database.** Gene expression profiles of DKK1 by the ovarian cancer disease state (A), FIGO stage (B), FIGO grade (C) and survival curve (D). Abbreviation: OSE, ovarian surface epithelium; FTE, fallopian tube epithelium; Mets, metastasis; Error bar is the median ± quantile. Kaplan-Meier analyses were conducted to estimate overall survival and disease-free survival. *p<0.05, ****p<0.0001.

Aberrant expression of DKK1 (Dickkopf-1) has been observed in numerous human cancers. To investigate the role of DKK1 in ovarian cancer development, we determined the DKK1 expression level in ovarian cancer tissue versus normal tissue in the CSIOVDB (Figure 1) and Oncomine (Figure S1) databases. Generally, DKK1 expression levels were decreased in ovarian tumors compared with normal ovarian surface epithelium (p=1.6E-8) and decreased in tumor stroma compared with normal stoma (p=3.8E-2) based on the CSIOVDB database (Figure 1A). DKK1 expression levels were also examined in different FIGO stages (Figure 1B) and FIGO grades (Figure 1C). Data indicated that compared with stage I patients, patients at advanced stage II (p=8.62E-4), III (p=6.76E-10) and IV (p=5.6E-6) exhibited lower expression levels of DKK1. Similarly, compared with grade 1 tumors, tumors classified as poorly differentiated grade 2 (p=6.22E-6) and 3 (p=1.58E-10) had lower expression of DKK1. Furthermore, Kaplan-Meier survival analyses were performed using CSIOVDB. Among patients with lower DKK1 expression, poorer overall survival and disease-free survival were observed (Figure 1D). Different datasets of the Oncomine database are presented as box plots (Figure S1) and tables (Supplement Table 1). Briefly, lower expression of DKK1 was found in ovarian carcinoma and ovarian serous adenocarcinomas than normal tissue based on the Bonome and Yoshihara subdatasets.

### CA125 downregulated DKK1 expression in ovarian cancer cells

**Figure 2:**
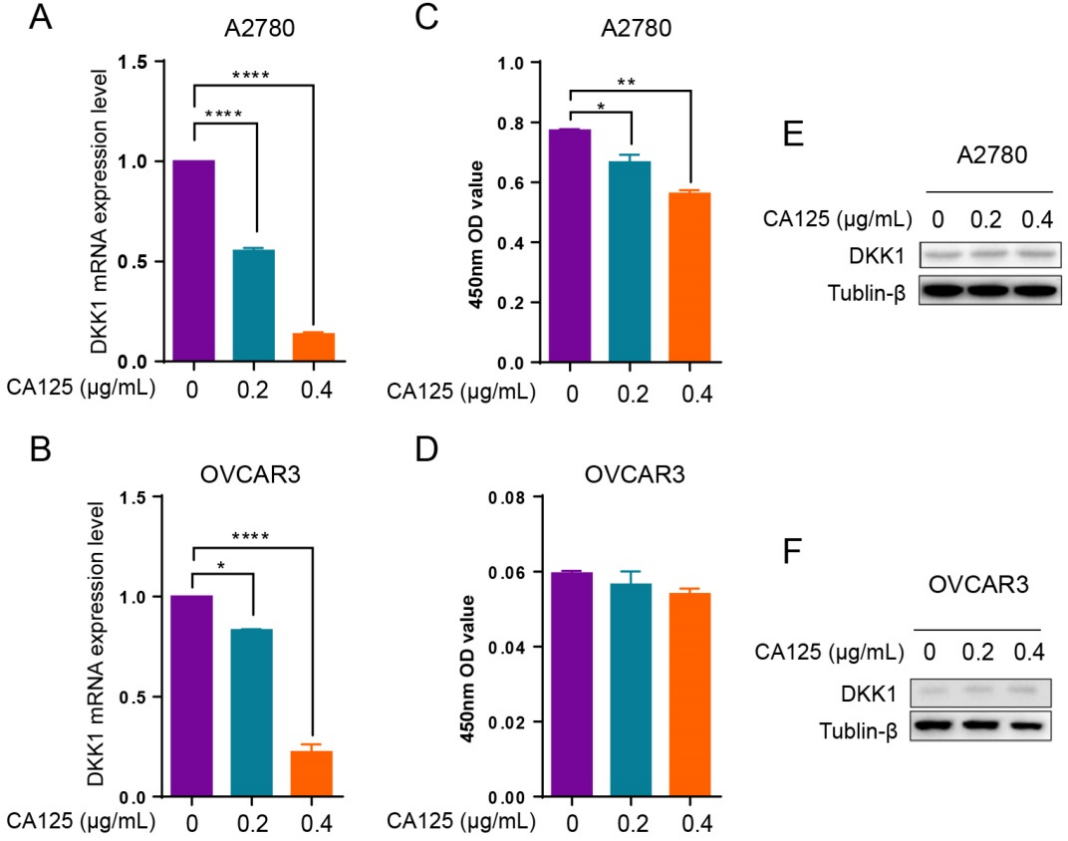
** CA125 downregulates DKK1 expression in ovarian cancer cells.** qPCR** (A-B)**, ELISA **(C-D)**, and Western blot **(E-F)** analysis of DKK1 mRNA levels in A2780 and OVCAR3 cells treated with CA125 at 0, 0.2. and 0.4 µg/mL for 48 h. The results represent the mean ± SD. *p<0.05, **p<0.01, ****p<0.0001.

The serum CA125 levels increased in most ovarian cancer patients. DKK1 decreased in ovarian cancer tissues at an advanced stage of disease progression. To determine the relationship between CA125 and DKK1 expression levels in ovarian cancer, we added CA125 to the culture medium of ovarian cancer cells. Since DKK1 is a secreted protein, the DKK1 mRNA and protein levels were detected by qPCR, Western blotting and ELISAs. The results demonstrated that the DKK1 mRNA levels were decreased with CA125 stimulation in A2780 and OVCAR3 cells (Figure 2A and B). Accordingly, ELISAs showed that the protein levels of DKK1 in the culture medium were decreased in two ovarian cancer cell lines (Figure 2C and D). However, the protein levels of DKK1 in OVCAR3 cells showed a decreased trend but no statistical significance. One potential reason is that OVCAR3 cells have higher expression levels of CA125, which may desensitize the cells to exogenous CA125. Western blot assays were conducted to examine the protein levels of DKK1 in the cell lysates, which showed nonsignificant changes (Figure 2E and F), indicating that the majority of DKK1 proteins were secreted into the culture medium.

### CA125 induced ovarian cancer cell migration by attenuating DKK1 expression

**Figure 3:**
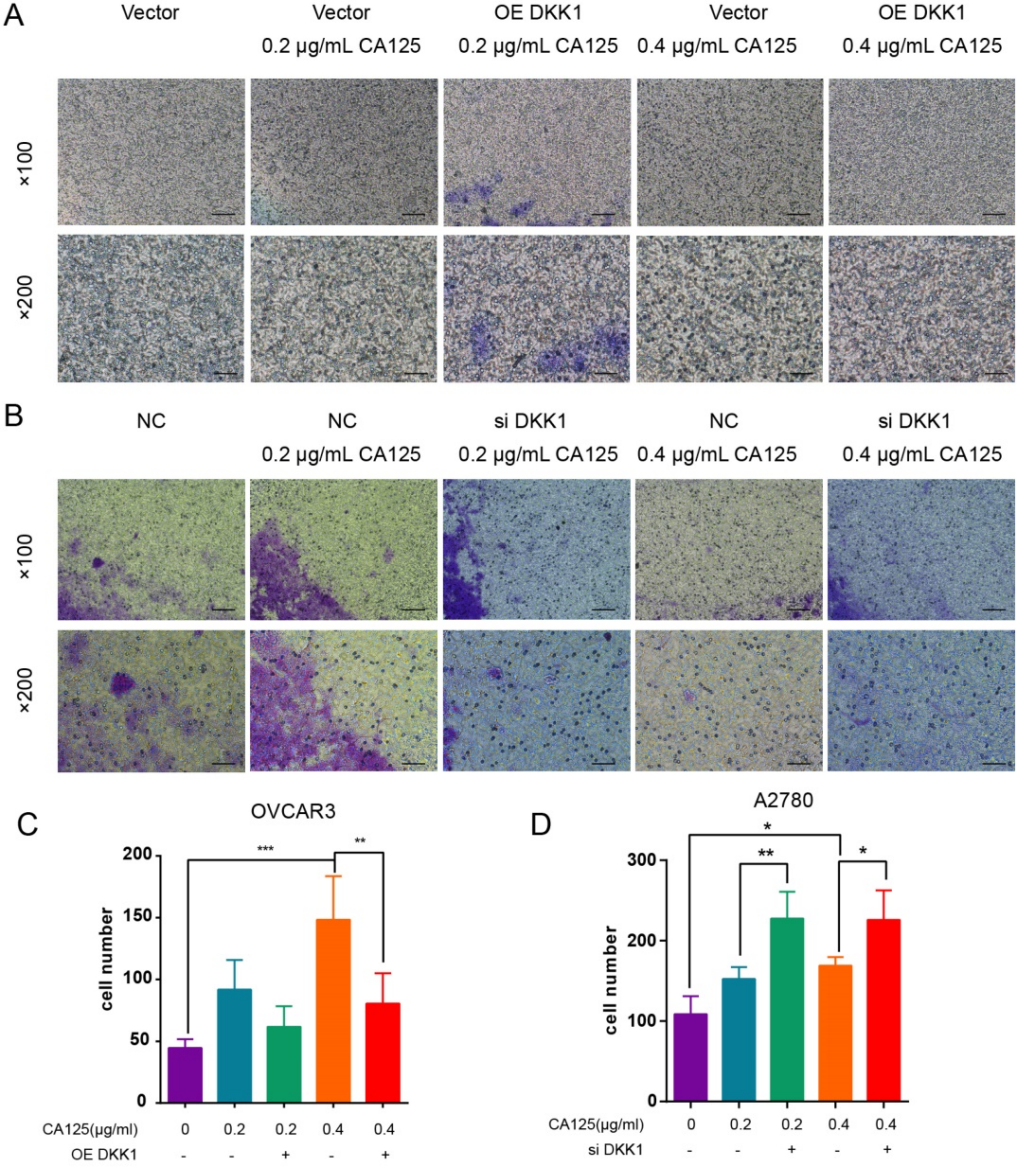
** CA125 induced ovarian cancer cell migration by attenuating DKK1 expression.** OVCAR3 cells **(A)** transfected with pEGFP-N1 vector and pEGFP-N1-DKK1 and A2780 **(B)** transfected with negative control (NC) and siDKK1 were subjected to Transwell migration assays and treated for 48 h with CA125 (scale bars=100 µm). **(C) (D)** Cell counts across the Transwell membrane. The statistical analyses were conducted with the number of invaded cells (indicated by red arrows). The results represent the mean ± SD. *p<0.05, **p<0.01, *** p<0.001.

We found that CA125 enhanced the migration of ovarian cancer cells 19. CA125 also downregulated the expression of DKK1 *in vitro.* To address the relationship between the CA125-mediated decrease in DKK1 expression and the enhanced migration of cells, we transfected the pEGFP-N1-DKK1 vector and siDKK1 into ovarian cancer cells to overexpress and knock down DKK1. The effects of DKK1 and CA125 on migration were observed in the Transwell assays. CA125 was observed to promote migration in both ovarian cancer cell lines. Figure 3A shows that pretreatment with the OE DKK1 vector significantly blocked cell migration, which was enhanced by CA125. Figure 3B shows that siDKK1 synergistically enhanced CA125 efficacy to promote ovarian cancer cell migration. Overall, these data indicated that CA125, through attenuating DKK1 expression, modulates ovarian cancer cell migration.

### Next-generation sequencing of the differentially expressed transcripts of ovarian cancer cells with CA125 stimulation

**Figure 4:**
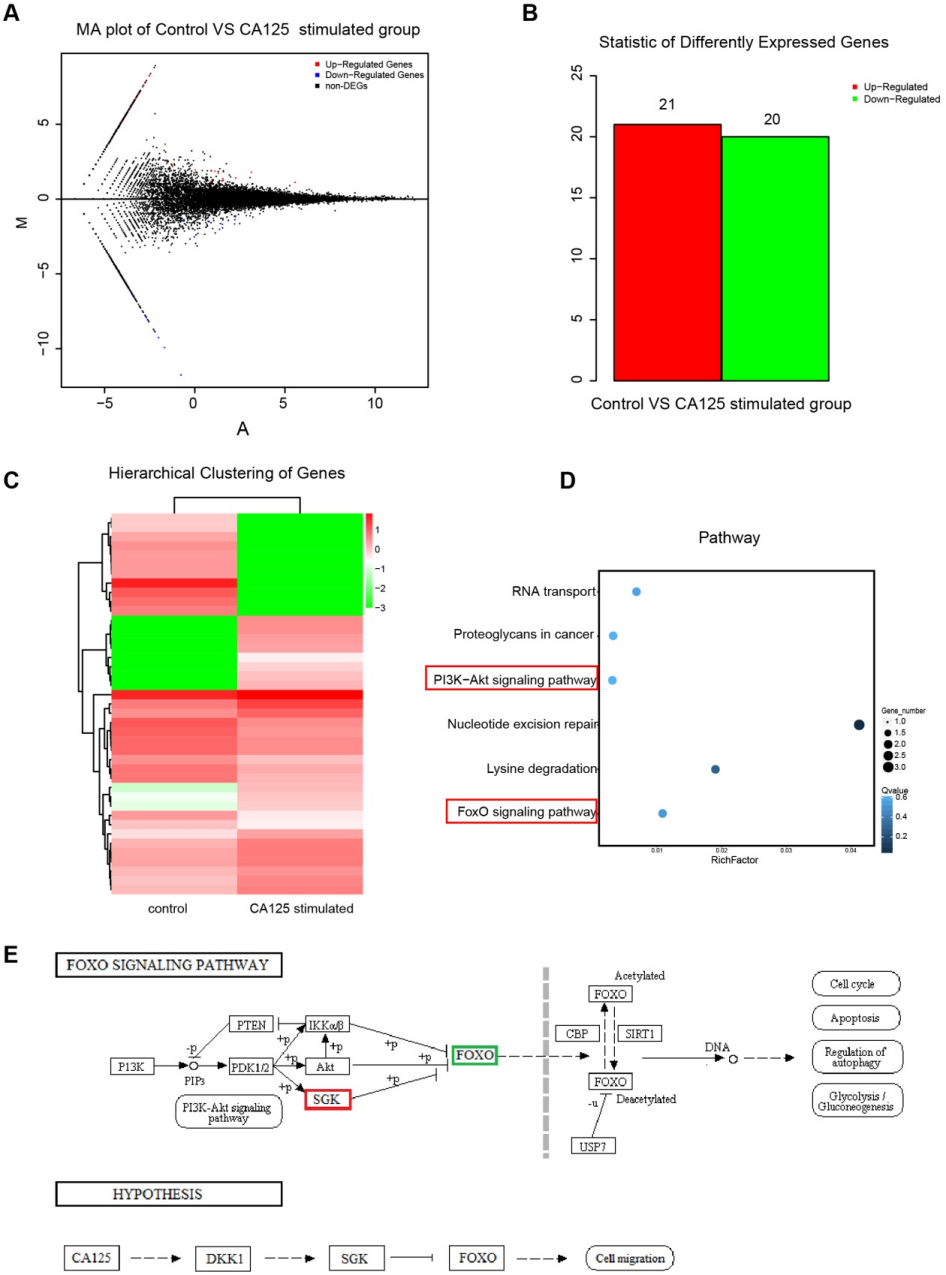
** Next-generation sequencing of the differential expression of transcripts of ovarian cancer cells with CA125 stimulation. (A)** Distribution of the differentially expressed transcripts in the MA plot. Red dots represent upregulated DEGs, blue dots represent downregulated DEGs, and black dots represent nondifferentially expressed genes. The abscissa is log2 (FPKM), and the ordinate log2 (FC). **(B)** Statistical histogram of DEGs. **(C)** Hierarchical clustering analysis of DEGs. **(D)** Enrichment scatter diagram of KEGG analyses of DEGs. The vertical axis represents the pathway, the horizontal axis represents the rich factor, the size of the point represents the number of DEGs in the pathway, and the color of the point corresponds to different Q-values. **(E)** Schematic diagram of the FOXO pathway and the hypothesis of the mechanism by which CA125 enhances cell migration.

To explore the mechanisms by which CA125 enhances ovarian cancer cell migration, we conducted next-generation sequencing to investigate the differential expression of transcripts in the CA125-stimulated and unstimulated cells. HISAT (Hierarchical Indexing for Spliced Alignment of Transcripts) was used to map the clean reads to the reference genome. A total of 25526 transcripts were identified. Poisson distribution analysis was used to model the read counts. Twenty-one upregulated and twenty downregulated differentially expressed genes (DEGs) were identified with the threshold of absolute value of fold change (FC)>1 and false discovery rate (FDR)≤0.001 (Figure 4A-B). The expression patterns of the sample mRNAs are presented as heat maps (Figure 4C). Kyoto Encyclopedia of Genes and Genomes (KEGG) enrichment analysis was used to assess the DEGs affecting both the PI3K/Akt pathway and FOXO pathway (Figure 4D). SGK3 (serum/glucocorticoid regulated kinase family member 3) (fold change, 6.11; FDR, 8.6E-7) was upregulated and FOXO3 (forkhead box O3) (fold change, -7.47; FDR, 3.14E-24) was downregulated in the CA125-stimulated group compared with the unstimulated group. A schematic diagram of the pathway was acquired based on the KEGG database, and a hypothesis was proposed to explain the mechanism by which CA125 enhances cell migration (Figure 4E). Namely, CA125 promotes the migration of ovarian cancer cells by reducing DKK1 expression and activating the SGK3/FOXO3 pathway.

### CA125 reduced DKK1 expression to activate the SGK3/FOXO3 pathway

**Figure 5:**
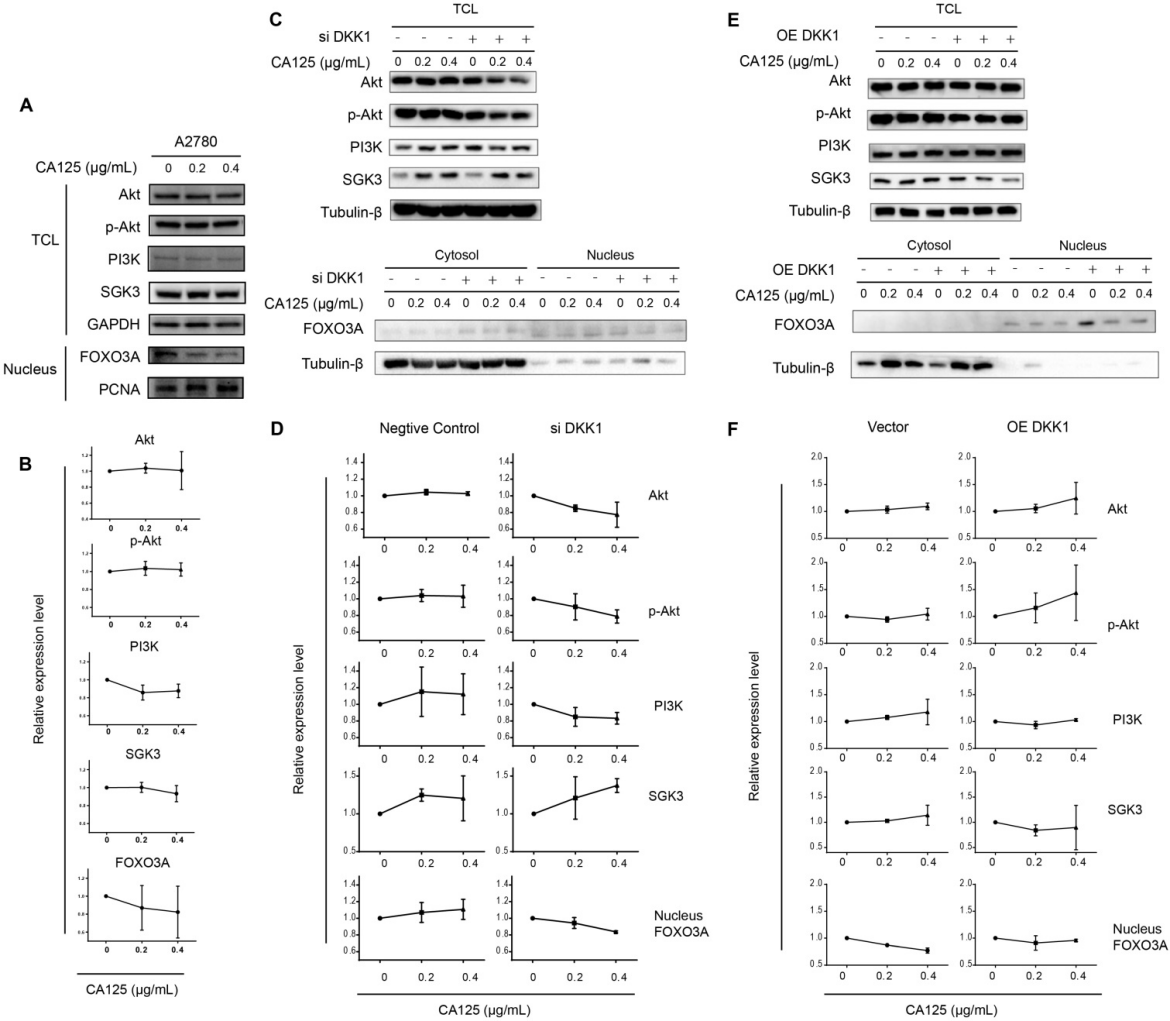
** CA125 reduces DKK1 expression to activate the SGK3/FOXO3 pathway. (A) (C) (E)** Western blots were performed to evaluate the expression of Akt, p-Akt, PI3K, and SGK3 in total cell lysates (TCL) and FOXO3 in cytoplasmic/nuclear protein extracts. GAPDH, Tubulin-β and PCNA were used as the experimental controls. **(B) (D) (F)** Relative protein expression of blots was quantified using densitometric analysis. The results represent the mean ± SD. There was no statistically significant difference.

SGKs (serum- and glucocorticoid-regulated kinases) are serine/threonine kinases that are similar to Akt; both phosphorylate FOXO3 and lead to the inhibition of FOXO3. FOXO3 primarily acts as a transcription factor in the nucleus, and phosphorylated FOXO3 binds to 14-3-3 protein and remains in the cytoplasm of cells 21. To validate the SGK3 and FOXO3 expression levels following CA125 stimulation and the hypothesis proposed based on the sequencing results, we performed Western blot and semiquantitative analyses of the optical density of these proteins. Because PI3K is common upstream of SGK3 and Akt, we first detected the expression levels of Akt, p-Akt, PI3K, SGK3 and FOXO3. The Akt, p-Akt, PI3K, and SGK3 protein levels in total cells were not significantly changed; however, the content of FOXO3 in the nucleus had a decreasing trend. These results were partially consistent with the sequencing results (Figure 5A-B).

Next, DKK1 was knocked down and overexpressed to determine the relationship between CA125-mediated reduction of DKK1 and activation of the SGK3/FOXO3 pathway. The expression of Akt, p-Akt, PI3K and SGK3 in the total cell lysates and the expression of FOXO3 in cytoplasmic/nuclear protein extracts were detected after transfection and stimulation with CA125 at 0, 0.2 and 0.4 μg/mL. The activation of the SGK3/FOXO3 pathway was estimated by the expression of SGK3 and the translocation of FOXO3. The expression of SGK3 had an increasing trend, and FOXO3 translocated from the nucleus to the cytosol in siDKK1 cells synergistically with CA125 stimulation (Figure 5C-D). OE DKK1 reversed the effect of CA125 on activating the SGK3/FOXO3 pathway (Figure 5E-F). The relative expression levels of total SGK3 or nuclear FOXO3 were not significantly changed in the OE DKK1 cells with CA125 stimulation. Interestingly, the expression of Akt, p-Akt and PI3K in the siDKK1 cells was decreased after CA125 stimulation. One possible reason is that SGK3 and Akt share a large number of substrates, and activated downstream substrate feedback inhibits Akt, p-Akt and PI3K.

### Anti-MSLN blockade of CA125 reduced the decrease in DKK1 and initiated apoptosis

**Figure 6:**
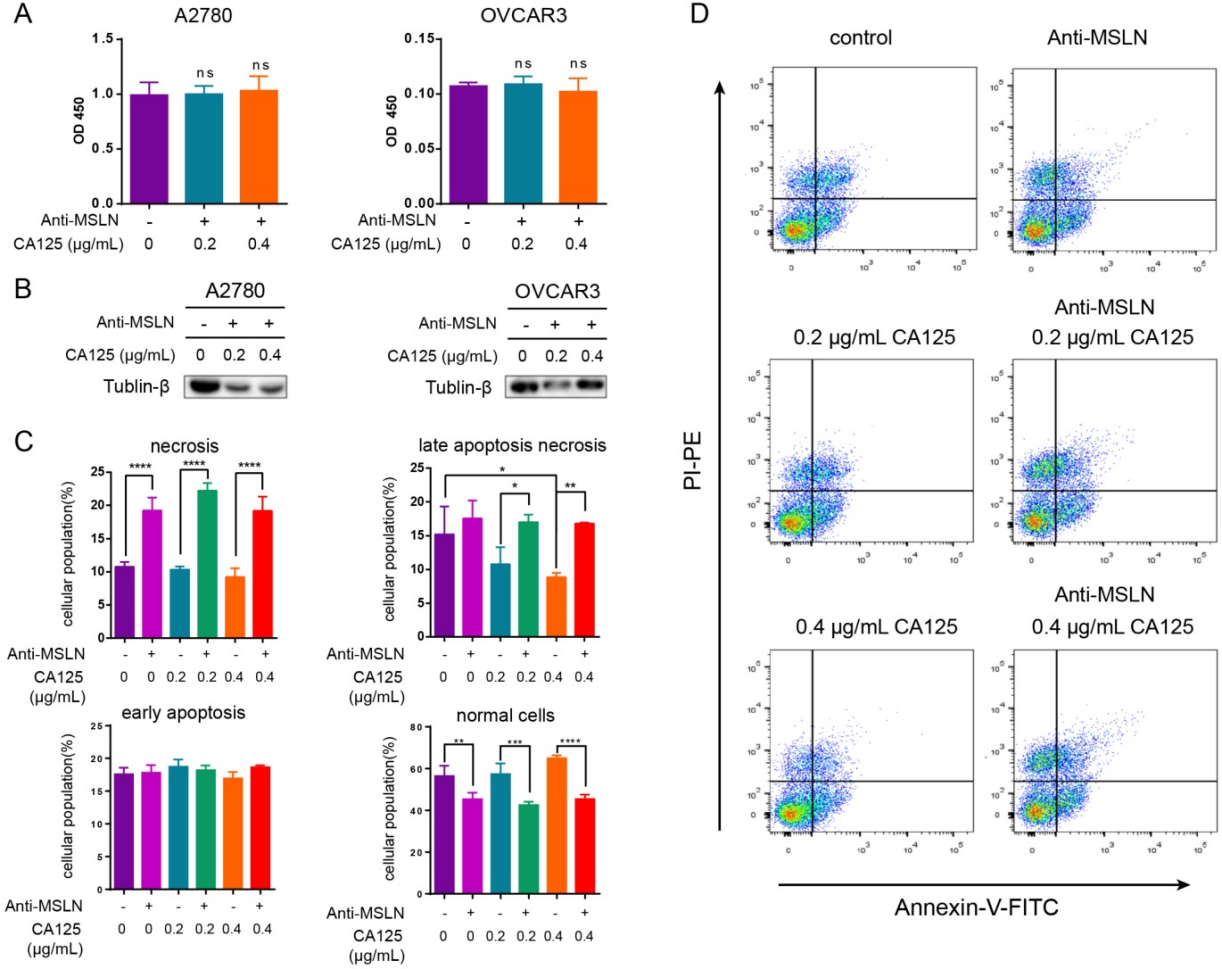
** Anti-MSLN blockade of CA125 reduced the decrease in DKK1 and initiated apoptosis. (A)** Expression of DKK1 was determined by ELISAs. **(B)** The expression levels of Tubulin-β in total cell lysates were determined by Western blots. **(C-D)** Flow cytometric analysis of OVCAR3 cells treated with and without anti-MSLN or CA125. Statistical analysis of the gated cells and representative images are shown. The results represent the mean ± SD. *p<0.05, **p<0.01, ***p<0.001, ****p<0.0001, ns, not significant.

MSLN is expressed on the surface of mesothelial cells, mesothelioma and ovarian cancer cells. To date, CA125 is the only known binding partner that interacts with MSLN 22. We hypothesized that the effect of CA125 on ovarian cancer cells was induced by the binding of CA125 to MSLN. To block this effect, we added anti-MSLN and CA125 to the cell culture medium. DKK1 protein expression did not change (p > 0.05) in two ovarian cancer cell lines stimulated with CA125 and anti-MSLN compared to the unstimulated cell line (Figure 6A). Dramatically, the cells treated with anti-MSLN showed a decreasing tendency to proliferate. The expression levels of Tubulin-β in total lysates were decreased by adding anti-MSLN and CA125 (Figure 6B). Subsequently, apoptosis was evaluated by Annexin/PI dual staining followed by flow cytometry to investigate the effect of CA125 and anti-MSLN. Cells were gated into four groups by staining according to the standard method 23. Figure 6C-D shows that CA125 significantly reduced late apoptotic necrosis at high concentrations (p<0.05). However, with the addition of anti-MSLN, necrosis and late apoptotic necrosis were both increased, particularly in cells with CA125 stimulation. Correspondingly, anti-MSLN dramatically reduced the number of viable cells.

### CA125 facilitated ovarian cancer metastasis *in vivo*

**Figure 7:**
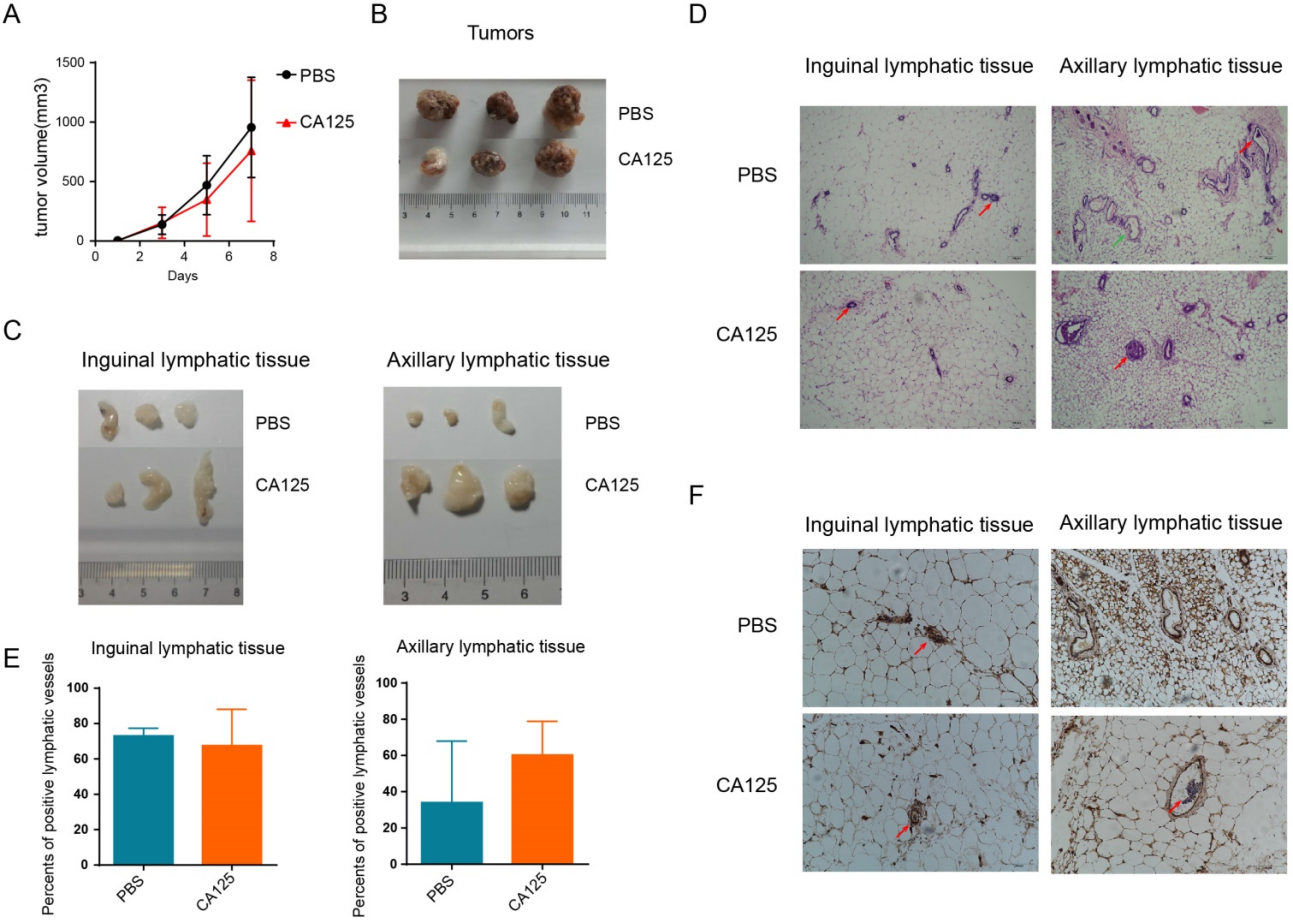
** CA125 facilitates ovarian cancer metastasis *in vivo*. (A)** The effect of CA125 on tumor growth was monitored by measuring tumor volume consecutively. Individual tumor images **(B)** and lymphatic tissue images **(C)** were taken after the mice were sacrificed. **(D)** Representative micrographs of hematoxylin and eosin (H&E) staining (scale bars=100 µm). Red arrows indicate metastases. Green arrows indicate normal lymphatic vessels. **(E)** The percentages of positive lymphatic vessels in the inguinal and axillary lymphatic tissues. The results represent the mean ± SD. **(F)** LYVE-1 histochemical staining of inguinal and axillary lymphatic tissues. The red arrow shows the tumor cells in the lymphatic vessel (scale bars=100 µm).

Our previous studies demonstrated that CA125 significantly promoted cell migration *in vitro* 19. To further confirm the role of CA125 in metastasis, we subcutaneously inoculated A2780 ovarian cancer cells expressing low CA125 levels into NOD-SCID-IL2rg mice. PBS or CA125 was injected via the tail vein once when the subcutaneous tumors were palpable (about 20 mm^3^). The tumor volumes were measured consecutively (Figure 7A), and the tumor images were taken after the mice were sacrificed (Figure 7B). Taken together, these results indicate that tumor volumes were not significantly different after CA125 injection compared with PBS injection. However, the inguinal and axillary lymphatic tissues were enlarged by CA125 injection (Figure 7C). Accordingly, tumor metastasis in the lymphatic vessels was observed with H&E staining of the lymphatic tissues (Figure 7 D). The lymphatic vessels with tumor cells were recorded as positive lymphatic vessels, and normal lymphatic vessels were recorded as negative lymphatic vessels. The proportion of positive lymphatic vessels in the inguinal and axillary lymphatic tissues was determined (Figure 7E). The metastases were similar in the inguinal lymphatic tissues in both the CA125-stimulated and PBS groups. However, the proportion of positive lymphatic vessels in the axillary lymphatic tissues increased in the CA125-stimulated group, indicating that CA125 induced metastasis to axillary lymphatic tissues *in vivo*. One potential reason is that the tumor cells were injected near the hind limbs; thus, the cells were more likely to invade the inguinal lymphatic tissues. Figure 7F is a typical histochemical staining picture of lymphatic vessel endothelial hyaluronan receptor-1 (LYVE-1). Lymphatic vessel endothelial cells in the lymphatic vessels were stained for LYVE-1.

### Anti-MSLN inhibited tumorigenesis and metastasis facilitated by CA125

**Figure 8:**
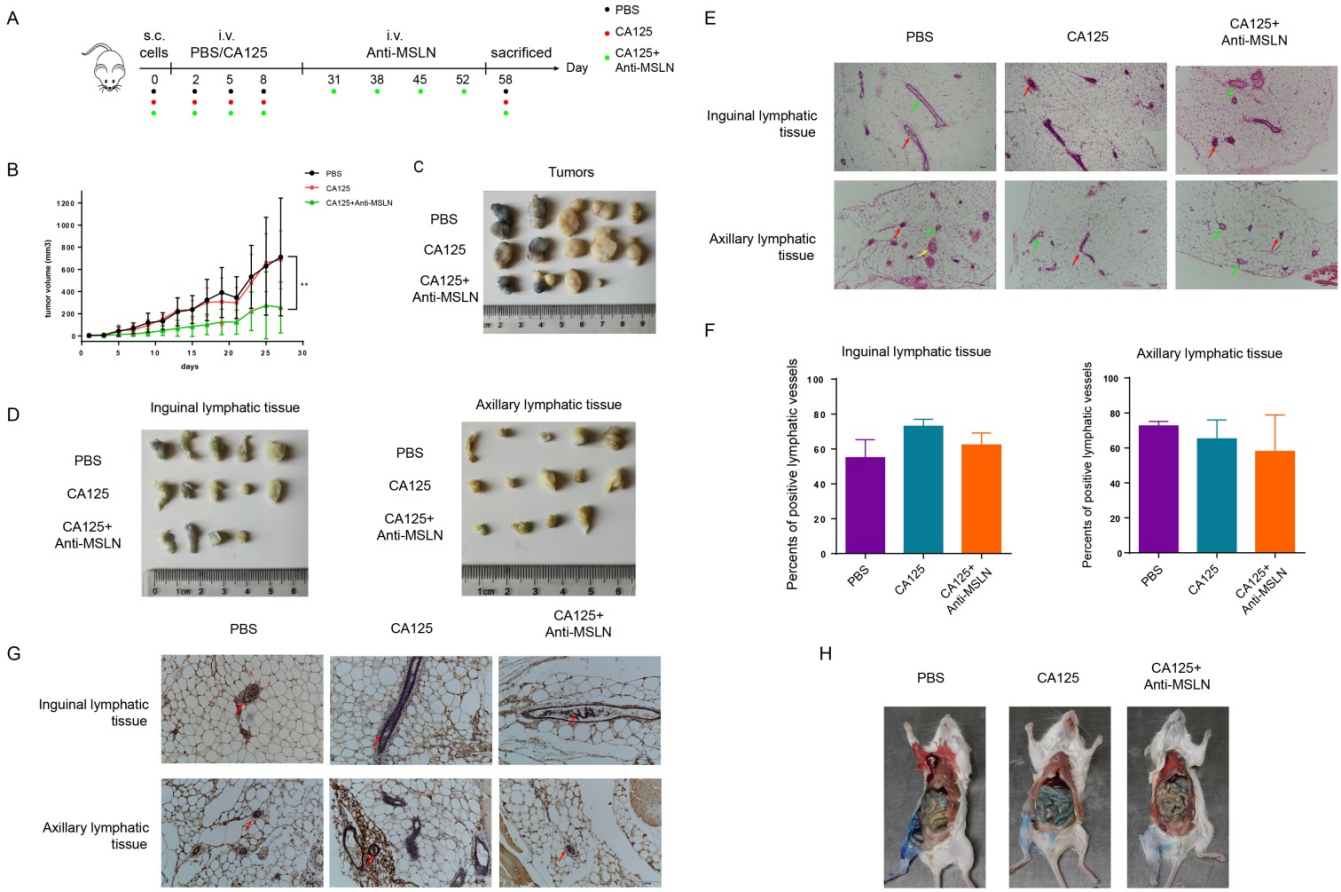
** Anti-MSLN inhibited tumorigenesis and metastasis facilitated by CA125. (A)** Schematic diagram of therapeutic strategies of anti-MSLN. **(B)** The therapeutic effect of anti-MSLN on tumor growth was monitored by consecutively measuring tumor volume. Individual tumor images **(C)** and lymphatic tissue images **(D)** were taken after the mice were sacrificed.** (E)** Representative micrographs of H&E staining (scale bars=100 µm). Red arrows indicate metastases. Green arrows indicate normal lymphatic vessels. Yellow arrows indicate the capillary.** (F)** Statistics of the percentages of positive lymphatic vessels in the inguinal and axillary lymphatic tissues. The results represent the mean ± SD. **(G)** LYVE-1 histochemical staining of inguinal and axillary lymphatic tissues. The red arrow shows the tumor cells in the lymphatic vessel (scale bars=100 µm).** (H)** Evan's blue lymphangiogram was used to evaluate tumor metastasis. Two-way ANOVA tests were used to compare the differences in tumor volume. The results represent the mean ± SD. **p<0.01.

Next, the therapeutic effects of anti-MSLN on ovarian cancer growth and metastasis were estimated *in vivo*. OVCAR3 cells expressing high levels of CA125 were chosen to evaluate the therapeutic effect. CA125 and PBS were injected in triplicate before four therapies followed, as the schematic diagram shows (Figure 8A). Similar to prior results, CA125 had no effect on tumor volume. However, anti-MSLN significantly reduced the tumor volume compared with that of the CA125 and PBS groups (Figure 8B-C). Accordingly, the lymphatic tissues were stripped, and the proportion of positive lymphatic vessels in the lymphatic tissues was determined according to H&E staining (Figure 8D-F). Figure 8G shown lymphatic vessel endothelial cells in the lymphatic vessels were stained for LYVE-1. The proportion of positive lymphatic vessels in the anti-MSLN group exhibited a downward trend. Evan's blue lymphangiogram was also used to evaluate tumor metastasis. The mesenteric tissues of mice injected with CA125 were hyperchromatic and were roughly eliminated when the mice were injected with anti-MSLN (Figure 8H). In brief, anti-MSLN treatment reduced tumorigenesis and metastasis.

## Discussion

DKK1 is a secreted glycoprotein that shares a binding receptor with Wnt to LRP5/6 (low-density lipoprotein receptor-related protein 5/6) 24. In the canonical Wnt pathway, DKK1 can block Wnt binding to the receptor and induce the release of Axin, therefore resulting in β-catenin degradation and translocation to the nucleus 25. In adults, DKK1 is implicated in cancer. In prostate cancer, elevated DKK1 expression is an early event, but as cancer progresses, DKK1 expression declines, particularly in advanced bone metastases 26. In breast cancer cells, DKK1 inhibits migration and invasion though suppression of the β-catenin/MMP7 pathway 27. These findings indicate that DKK1 may be involved in cancer migration and metastasis. In epithelial ovarian cancer, research has revealed that ten-eleven translocation 1 (TET1) inhibits cell migration and invasion by upregulating DKK1 28, which is partly consistent with our study. To dissect the effect of DKK1 in ovarian cancer, we analyzed several public databases. The DKK1 expression levels were downregulated in ovarian cancer tumors and correlated with FIGO stages, grades and disease-free survival. However, the serum CA125 level, as a tumor marker, was mostly elevated in ovarian cancer patients. Our data demonstrated that the DKK1 expression level was downregulated by elevated CA125 and was related to migration induced by CA125 in ovarian cancer cells *in vitro*. Traditionally, DKK1 is considered an inhibitor of the Wnt/β-catenin pathway. However, our data revealed that although CA125 strongly decreased the expression levels of DKK1, the Wnt/β-catenin pathway was inactive in ovarian cancer cells.

In addition to the precise dysregulation of the Wnt signaling pathway, DKK1 is related to the PI3K/Akt pathway. In a recent study by Orestis Lyros et al., knocking down DKK1 significantly activated Akt phosphorylation independent of the Wnt pathway 29. It has been demonstrated that DKK1 binds to cytoskeleton-associated protein 4 (CKAP4) with high affinity, leading to the activation of Akt by forming a complex between CKAP4 and PI3K, resulting in the proliferation of normal cells and cancer cells 30. Our data support the theory that DKK1 is related to pathways other than the Wnt/β-catenin pathway.

The serum- and glucocorticoid-regulated kinase (SGK) family, composed of three isoforms, is similar to Akt kinases, sharing similar structure, substrate specificity and function with Akt; both are activated downstream of the PI3K pathway 31,32. Increasing studies have shown that SGK3 is dysregulated in cancer in an Akt-independent manner 33,34. In prostate cancer, SGK3 is an androgen receptor transcriptional target and promotes cancer cell proliferation 35. Forkhead box O (FOXO) transcription factor can regulate cells by modulating apoptosis 36, the cell cycle 37, DNA repair 38 and migration 39. SGK3 recognizes the same substrate phosphorylation motif as Akt, RXRXXS/T (R indicates arginine, X indicates any amino acid, and S/T indicates serine/threonine), and phosphorylates FOXO3 in preferred sites 40. Phosphorylated FOXO3 increases binding with 14-3-3 proteins. The binding prevents FOXO3 nuclear reimport, leading to the translocation of FOXO3 from the nucleus to the cytoplasm 42. Our data suggest that CA125 activates the SGK3/FOXO3 pathway by reducing the expression of DKK1. The translocation from the nucleus to the cytosol of FOXO3 was observed under CA125 stimulation, which operated in synergy with siDKK1 and was reversed by OE DKK1. Notably, SGK3 and Akt share overlapping substrates that modulate feedback inhibition 45. Akt and p-Akt were feedback inhibited under the joint effects of CA125 and siDKK1.

Mesothelin (MSLN) was first described in the early 1990s by Ira Pastan and Mark Willingham 46. This protein is expressed on the surface of human ovarian carcinoma cells and normal mesothelium. 47. MSLN was estimated to be strongly overexpressed in nearly one-third of human malignancies and is therefore a very important target for immunotherapy 49,50. MSLN is highly expressed in 70% of ovarian cancers, including up to 100% of serous papillary ovarian cancers 51,52. Initial experiments suggested that mesothelin is involved in cell adhesion events 48. In 2004, researchers demonstrated the binding of MSLN to CA125 by flow cytometry as well as immunoprecipitation and found that CA125 and MSLN are co-expressed in advanced ovarian cancer. The binding of CA125 and membrane MSLN might contribute to the metastasis of ovarian cancer by initiating cell attachment 53. Mesothelin-CA125 binding has been proven to be a high affinity, N-glycan-dependent interaction that increases cell adhesion 54. The binding occurs with a strong affinity with an apparent Kd of 5-10 nM, and the N-linked oligosaccharides of CA125 and the N-terminus of mesothelin are necessary in this binding 54,55. A study proved that CA125 has different mesothelin-binding abilities in patients with endometriosis and epithelial ovarian cancer. This mesothelin-binding ability was significantly higher in epithelial ovarian cancer patients than in patients with endometriosis 57. Thus, in OC patients, CA125 may have a strong affinity for binding to mesothelin in ovarian cancer cells to trigger further effects that enhance metastasis. Our data showed that anti-MSLN can block the reduction of DKK1 mediated by CA125 in ovarian cancer cells.

In addition, MSLN protects cells from drug-induced apoptosis and mediates drug resistance in cancer 58,59,60. In our studies, anti-MSLN was proven to induce apoptosis and necrosis of ovarian cancer cells. The possible mechanism of apoptosis is as follows: anti-MSLN can block CA125 from binding with mesothelin, which reverses the effect of CA125 on cell migration and apoptosis, and anti-MSLN inhibits the ability of mesothelin to protect cells from apoptosis.

**Figure 9:**
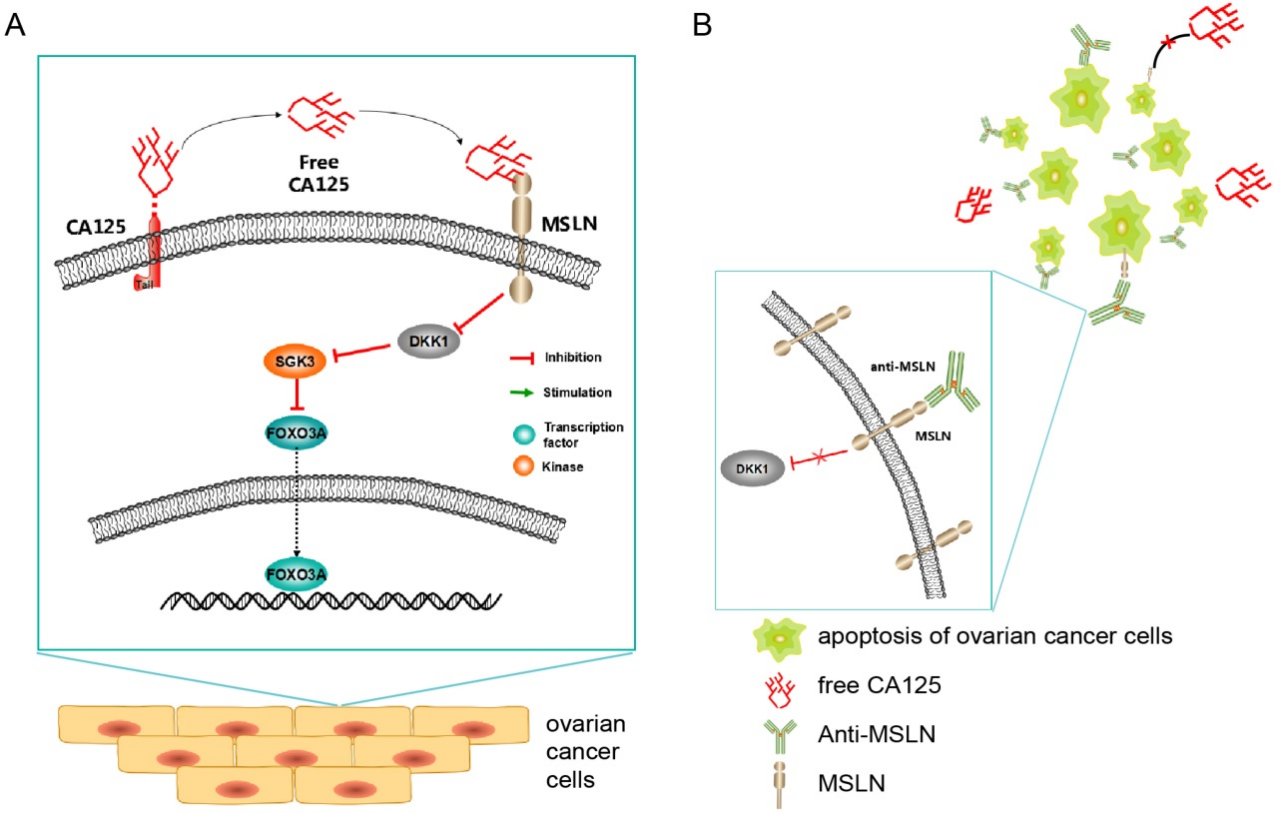
** Schematic diagram of the study. (A)** Schematic diagram of the potential mechanism of CA125-induced migration.** (B)** Schematic diagram of the potential therapeutic mechanism.

Schematic diagram (Figure 9) showing the potential mechanism of the anti-MSLN antibody blocking the effect of CA125 on inducing migration. High expression of CA125 on ovarian cancer cells results in release of the fragment free CA125, which binds to the mesothelin on the surface of ovarian cancer cells to reduce DKK1 expression. The reduction of DKK1 activated the SGK3/FOXO3 pathway to accelerate cell migration and tumor metastasis (Figure 9A). Once anti-MSLN was added, free-CA125 could no longer bind with ovarian cancer cells. The combination of anti-MSLN and ovarian cancer cells reversed migration and apoptosis (Figure 9B).

In tumor xenograft mice, CA125 was proven to promote the metastasis of ovarian cancer. Compared with those of the mice injected with PBS, the axillary lymphatic tissues of the mice injected with CA125 were enlarged, and the proportion of lymphatic vessels containing tumor cells was increased. These results suggested that CA125 can promote the metastasis of ovarian cancer in mice.

The therapeutic effects of anti-MSLN on ovarian cancer growth and metastasis were confirmed *in vivo*. The results showed that the tumors of mice injected with anti-MSLN were smaller than those injected with PBS or CA125, indicating that tumor growth was inhibited by anti-MSLN. At present, Evan's blue lymphatic tracer is often used in mice to assess tumor metastasis in mice 61. In our experiment, mesenteric drainage was increased in the CA125-treated mice but decreased in the mice treated with anti-MSLN. These results indicated that anti-MSLN can inhibit the growth and metastasis of ovarian cancer.

## Conclusion

In summary, the expression levels of DKK1 were downregulated in ovarian cancer patients and correlated with FIGO stage, grade and disease-free survival. Our data suggest that CA125 enhanced the migration and activated the SGK3/FOXO3 pathway by decreasing DKK1. Anti-MSLN can block the reduction of DKK1 mediated by CA125 and initiate apoptosis *in vitro*. *In vivo*, our data suggest that antibodies targeting mesothelin may offer a therapeutic option for ovarian cancer patients with high levels of serum CA125.

## Supplementary Material

Supplementary figures and tables.

## Figures and Tables

**Table 1:** Differentially expressed genes in pathway analysis

Pathway	Differentially expressed genes
RNA transport	RGPD6; EIF3CL
Proteoglycans in cancer	LUZP6; MTPN
PI3K-Akt signaling pathway	SGK3; FOXO3B
Nucleotide excision repair	GTF2H2C; BIVM-ERCC5; BIVM
Lysine degradation	LUZP6; MTPN
FoxO signaling pathway	SGK3; FOXO3B

## Abbreviations

OC: ovarian cancer
CA125: cancer antigen 125
MUC16: Mucin 16
DKK1: Dickkopf-1
SGK3: serum/glucocorticoid regulated kinase family member 3
FOXO3: forkhead box O3
MSLN: mesothelin
GAPDH: glyceraldehyde-3-phosphate dehydrogenase
PI3K: phosphatidylinositol-3 kinase
TET1: Ten-eleven translocation 1
LPR5/6: Low density lipoprotein receptor-related protein 5/6
CKAP4: cytoskeleton-associated protein 4
DEGs: differentially expressed genes
MPF: megakaryocyte potentiating factor
TNF: tumor necrosis factor
ELISA: enzyme-Linked Immuno Sorbent Assay
OD: optical density
q-PCR: Quantitative Polymerase chain reaction
SDS-PAGE: Sodium dodecyl sulfate-Polyacrylamide gelelectrophoresis
PBS: Phosphate-buffered saline
siRNA: Small interfering RNA
OE: Over Expression
FC: fold change
FDR: False Discovery Rate
KEGG: Kyoto Encyclopedia of Genes and Genomes
FITC: fluorescein isothiocyanate
PI: Propidium Iodide
MW: molecular weight
