# Supplementary material for: Free CA125 promotes ovarian cancer cell migration and tumor metastasis by binding Mesothelin to reduce DKK1 expression and activate the SGK3/FOXO3 pathway
Source: Int J Biol Sci. 2021 Jan 14;17(2):574–88. doi: 10.7150/ijbs.52097 (PMC7893585; doi:10.7150/ijbs.52097)
Supplement: Supplementary file 1 — Supplementary figures and tables. [file ijbsv17p0574s1.pdf]

Supplement

Results

DKK1 is down-regulated in ovarian cancer tissue

DKK1 expression level was analyzed of ovarian cancer tissue versus normal tissue by Oncomine database. DKK1 were non-significantly change in ovarian endometrioid adenocarcinoma, ovarian mucinous adenocarcinoma and ovarian serous adenocarcinoma versus normal tissue based on Hendrix, TCGA, Adib, Lu datasets. The distribution of DKK1 expression in endometrial adenocarcinoma is quite different. However, lower expression levels of DKK1 were found in ovarian carcinoma and ovarian serous adenocarcinomas versus normal tissue based on Bonome and Yoshihara datasets. The data of median, extremum and percentile was listed in table 1 corresponding to figure 1.

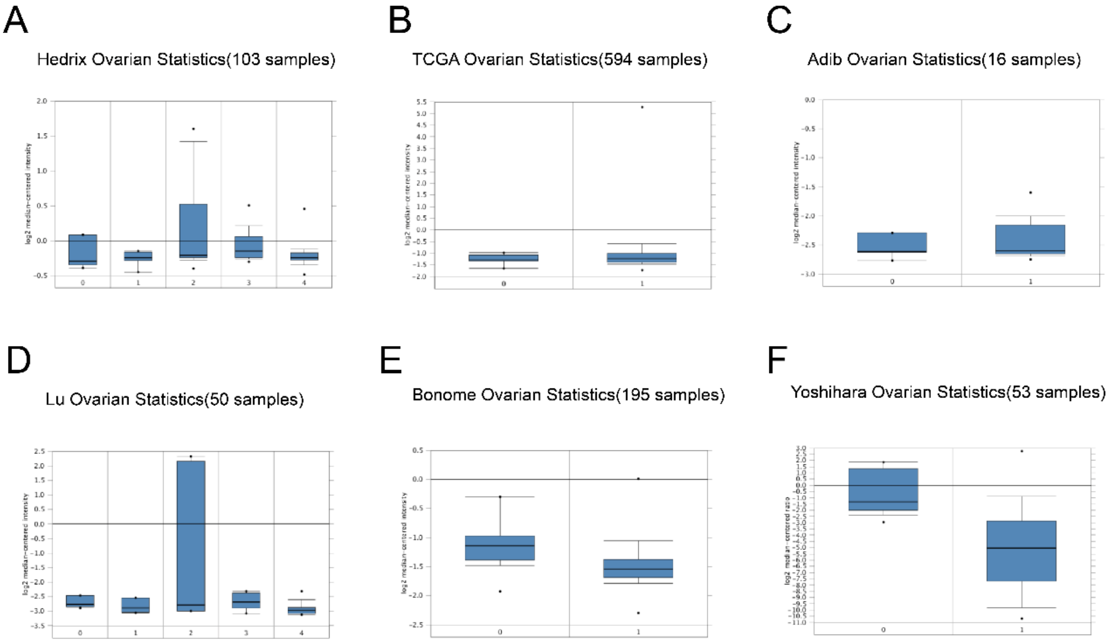

Figure S1. DKK1 mRNA expression analysis in ovarian cancer based on Oncomine Database. Box plots comparing specific DKK1 expression in normal and cancer tissue. **(A)** Hendrix (0, Normal tissue; 1, Ovarian Clear Cell Adenocarcinoma; 2, Ovarian Endometrioid Adenocarcinoma; 3, Ovarian Mucinous Adenocarcinoma; 4, Ovarian Serous Adenocarcinoma). **(B)** TCGA (0, Normal Ovarian; 1, Ovarian Serous Cystadenocarcinoma). **(C)** Adib (0, Normal Ovarian; 1, Ovarian Serous Adenocarcinoma). **(D)** Lu (0, Normal ovarian surface epithelium samples; 1, Ovarian Clear Cell Adenocarcinoma; 2, Ovarian Endometrioid Adenocarcinoma; 3, Ovarian Mucinous Adenocarcinoma; 4, Ovarian Serous Adenocarcinoma). **(E)** Bonome (Normal ovarian surface epithelium samples; 1, Ovarian carcinoma). **(F)** Yoshihara (Normal peritoneum samples; 1, Ovarian serous adenocarcinomas). Circles stand for outliers. The data was threshold by: p-value as 1E-8; fold change as 2.

Sample type	Sample number	Log2 median-centered intensity							reference
		Median	Maximum	Minimum	90th pecentile	10th pecentile	75th pecentile	25th pecentile	

**Hendrix Ovarian**									
**Normal tissue**	4	-0.294	0.082	-0.391	0.082	-0.391	0.082	-0.348	[1]
**Ovarian Clear Cell Adenocarcinoma**	8	-0.244	-0.151	-0.455	-0.151	-0.455	-0.164	-0.289	
**Ovarian Endometrioid Adenocarcinoma**	37	-0.211	1.6	-0.404	1.42	-0.276	0.521	-0.249	
**Ovarian Mucinous Adenocarcinoma**	13	-0.151	0.505	0.305	0.215	-0.266	0.055	-0.249	
**Ovarian Serous Adenocarcinoma**	41	-0.249	0.450	-0.487	-0.121	-0.347	-0.179	-0.28	
**TCGA Ovarian**									
**Normal Ovarian**	8	-1.294	-0.976	-1.675	-0.976	-1.657	-1.073	-1.352	TCGA (http://tcga-data.nci.nih.gov/tcga/)
**Ovarian Serous Cystadenocarcinoma**	586	-1.228	5.275	-1.725	-0.578	-1.471	-1	-1.381	
**Adib Ovarian**									
**Normal Ovarian**	4	-2.617	-2.289	-2.769	-2.289	-2.769	-2.289	-2.626	[2]
**Ovarian Serous Adenocarcinoma**	12	-2.597	-1.594	-2..743	-1.998	-2.696	-2.16	-2.651	
**Lu Ovarian**									
**Normal ovarian surface epithelium samples**	5	-2.761	-2.463	-2.895	-2.463	-2.895	-2.463	-2.848	[3]
**Ovarian Clear Cell Adenocarcinoma**	7	-2.888	-2.534	-3.065	-2.534	-3.065	-2.534	-3.03	
**Ovarian Endometrioid Adenocarcinoma**	9	-2.788	2.329	-3	2.329	-3	2.158	-2.985	
**Ovarian Mucinous Adenocarcinoma**	9	-2.69	-2.312	-3.085	-2.312	-3.085	-2.372	-2.883	
**Ovarian Serous Adenocarcinoma**	20	-3.065	-2.306	-3.121	-2.605	-3.11	-2.873	-3.074	
**Bonome Ovarian**									
**Normal ovarian surface epithelium samples**	10	-1.136	-0.3	-1.927	-0.3	-1.481	-0.974	-1.388	[4]
**Ovarian carcinoma**	185	-1.54	0.009	-2.297	-1.061	-1.79	-1.375	-1.692	
**Yoshihara Ovarian**									

**Normal peritoneum samples**	10	-1.334	1.871	-2.969	1.871	-2.37	1.314	-2.009	[5]
**Ovarian serous adenocarcinomas**	27	-5.028	2.741	-10.705	-0.874	-9.815	-2.849	-7.703	

Table S1. The changes of DKK1 mRNA expression level between different datasets in oncomine database. The data was threshold by: p-value as 1E-8; fold change as 2.

Confirmation of transfection availability

Cell DKK1 expression levels were determined in four ovarian cancer cell lines by q-PCR (Figure S2 A). Briefly, DKK1 is high-expressed in A2780, and low-expressed in OVCAR3. OVCAR3 cells were used to transfect the control pEGFP-N1 vector and pEGFP-N1-DKK1 vector. q-PCR, ELISA, Western Blot assays results were shown in Figure S2 B, DKK1 expression were up regulated in OE DKK1 group compared with control vector group. A2780 cells were used to transfect the negative control (NC) and siDKK1. Relative DKK1 mRNA expression levels after transfection of a series of siRNA were shown in Figure S2 C. Alignment siRNA2 was chosen to carry out the following experiments. Figure S2 D shown the q-PCR, ELISA, Western Blot assays results by using siRNA2. The expression levels of DKK1 were decreased by transfecting siDKK1 compared with NC.

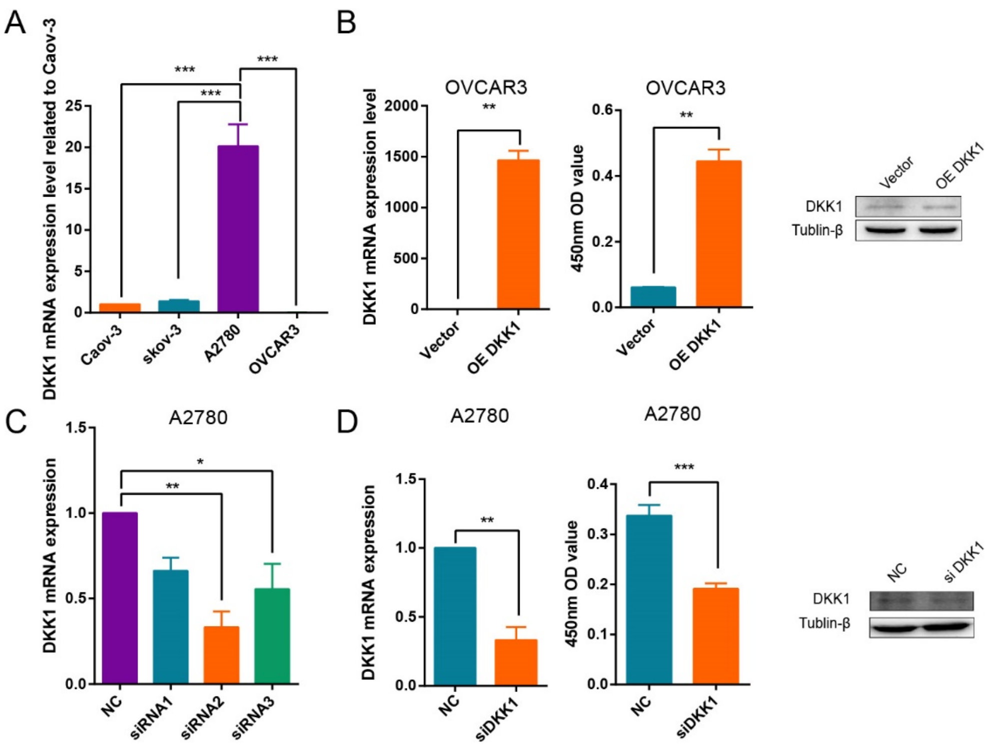

Figure S2. Confirmation of transfection availability. (A) DKK1 mRNA expression levels in a panel of ovarian cancer cell lines were determined by q-PCR. **(B and D)** DKK1 mRNA and protein expression levels in OVCAR3 and A2780 were analyzed by q-PCR, ELISA, Western

Blot assays. GAPDH were used as the q-PCR experiment control. Tublin-β were used as the Western experiment control. **(C)** DKK1 mRNA expression levels in A2780 were analyzed by q-PCR. The results represent the means ± SD. *p<0.05, **p<0.01, ***p<0.001, ****p<0.0001.

CA125 stimulation decreased DKK1 expression but did not inactivate Wnt/β-Catenin pathway

DKK1 (Dickkopf-1) is an inhibitor of Wnt/β-Catenin pathway, which can induce the release of Axin and result in β-catenin degradation and translocation to the nucleus. Based on the results that CA125 down-regulated the expression of DKK1 in ovarian cancer cells. The activation of Wnt/β-Catenin pathway after CA125 stimulation was detected through the nucleus/cytosol fractionation experiments and the expression of the downstream gene of Wnt/β-Catenin pathway. Figure S3 A shown that neither significantly changes were observed of the DKK1 expression levels nor the cytosol-nucleus translocation under CA125 stimulation in both ovarian cancer cell lines. Consistent with these results, q-PCR analysis showed that AXIN2 mRNA expression levels were no significantly changes in both ovarian cancer cell lines. In summary, although CA125 remarkably decreased the mRNA and protein expression of DKK1, the Wnt/β-Catenin pathway was inactive.

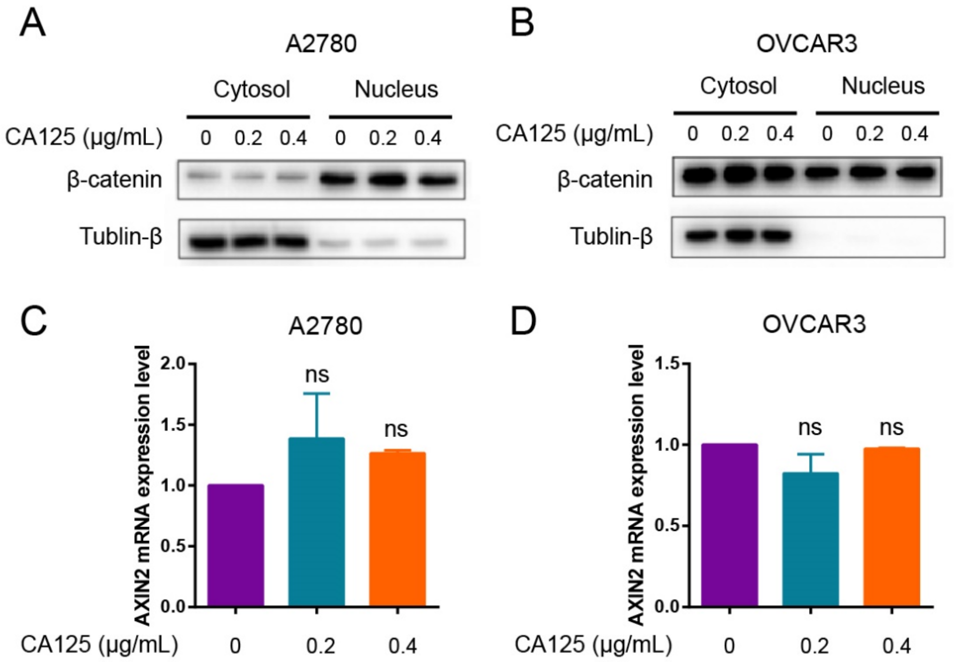

Figure S3. CA125 stimulation decreased DKK1 expression but did not inactivate Wnt/β-Catenin pathway. Cells were pretreated with CA125 for 48 h before detection. **(A-B)** The nucleus/cytosol fractionation lysates were immunoblotted with corresponding antibodies. The β-catenin expression levels were determined by Western blot in A2780 and OVCAR3 cells. Tublin-β were used as the experiment control. **(C-D)** The relative mRNA expression levels of AXIN2 were determined by q-PCR in A2780 and OVCAR3 cells. GAPDH were used as the experiment control. The results represent the means ± SD. ns, non-significance.

Anti-MSLN initiates apoptosis in A2780 cell line.

A2780 cell line also selected to investigate the role of Anti-MSLN and CA125 in ovarian cancer cells apoptosis. Figure S4 shown comparable results in A2780 as Figure 6 C-D. Briefly, cells necrosis significantly increased with CA125 and Anti-MSLN compared to individual CA125 stimulation.

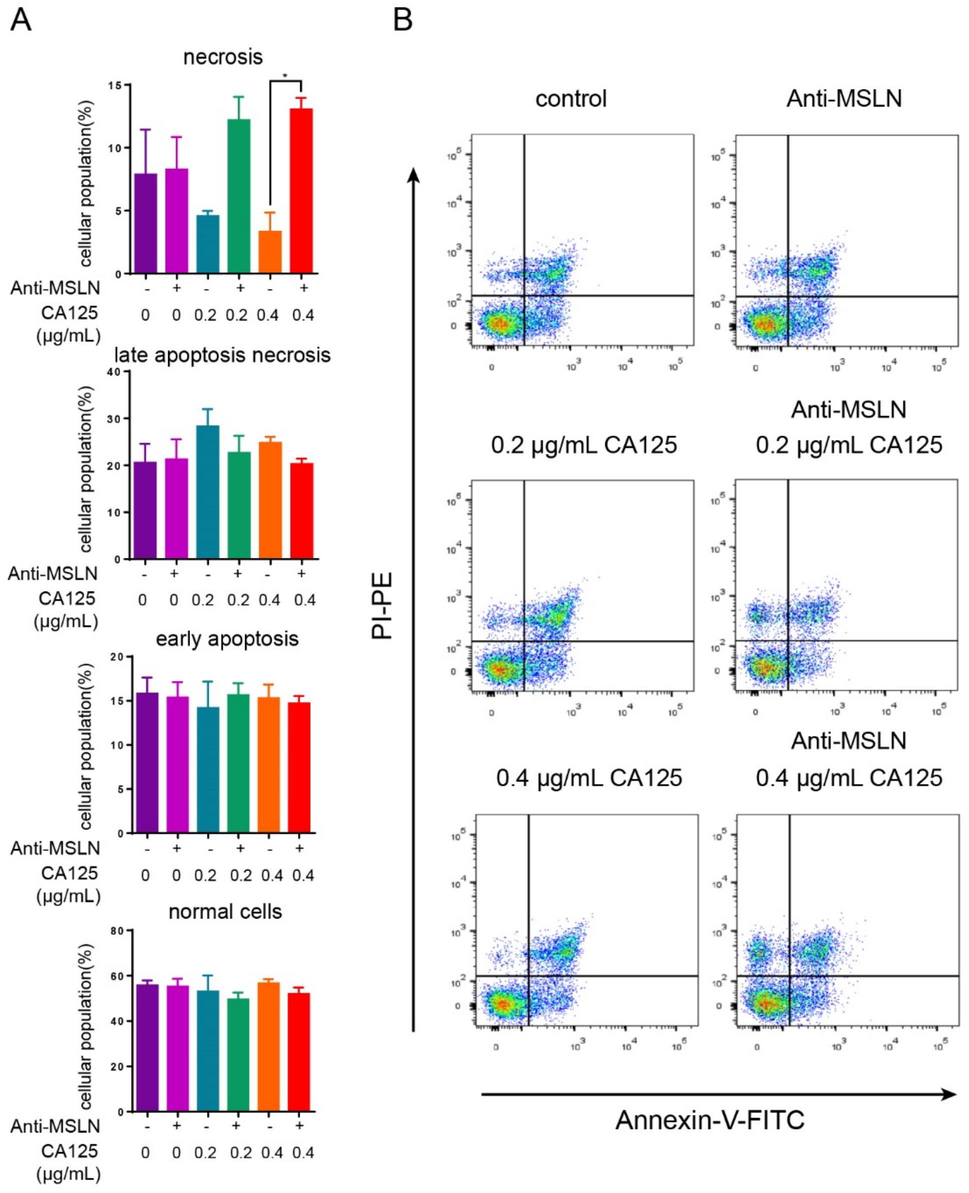

Figure S4. Anti-MSLN initiates apoptosis in A2780 cell line. Flow cytometric analysis of A2780 cells treated with and without Anti-MSLN or CA125. Statistical analysis of the gated cells**(A)** and representative image**(B)** were shown. The results represent the means ± SD. *$p<0.05$.

Reference

[1] Hendrix N D, Wu R, Kuick R, et al. Fibroblast growth factor 9 has oncogenic activity and is a downstream target of Wnt signaling in ovarian endometrioid adenocarcinomas[J]. Cancer Res, 2006,66(3):1354-1362.

[2] Adib T R, Henderson S, Perrett C, et al. Predicting biomarkers for ovarian cancer using gene-expression microarrays[J]. Br J Cancer, 2004,90(3):686-692.

[3] Lu K H, Patterson A P, Wang L, et al. Selection of potential markers for epithelial ovarian cancer with gene expression arrays and recursive descent partition analysis[J]. Clin Cancer Res, 2004,10(10):3291-3300.

[4] Bonome T, Levine D A, Shih J, et al. A gene signature predicting for survival in suboptimally debulked patients with ovarian cancer[J]. Cancer Res, 2008,68(13):5478-5486.

[5] Yoshihara K, Tajima A, Komata D, et al. Gene expression profiling of advanced-stage serous ovarian cancers distinguishes novel subclasses and implicates ZEB2 in tumor progression and prognosis[J]. Cancer Sci, 2009,100(8):1421-1428.
